# Crystallization and crystal-packing studies of *Chlorella* virus deoxyuridine triphosphatase

**DOI:** 10.1107/S1744309109034459

**Published:** 2009-09-25

**Authors:** Kohei Homma, Hideaki Moriyama

**Affiliations:** a Department of Chemistry, University of Nebraska-Lincoln, NE 68583-0304, USA; b Center for Biotechnology, University of Nebraska-Lincoln, NE 68588-0666, USA; c School of Biological Sciences, University of Nebraska-Lincoln, NE 68583-0118, USA

**Keywords:** *Chlorella* virus, *Chlorella*, dUTPases

## Abstract

The 141-amino-acid deoxyuridine triphosphatase (dUTPase) from the algal *Chlorella* virus IL-3A and its Glu81Ser/Thr84Arg-mutant derivative Mu-22 were crystallized using the hanging-drop vapor-diffusion method at 298 K with polyethylene glycol as the precipitant. An apo IL-3A dUTPase with an amino-terminal T7 epitope tag and a carboxy-terminal histidine tag yielded cubic *P*2_1_3 crystals with unit-cell parameter *a* = 106.65 Å. In the presence of dUDP, the enzyme produced thin stacked orthorhombic *P*222 crystals with unit-cell parameters *a* = 81.0, *b* = 96.2, *c* = 132.8 Å. T7-histidine-tagged Mu-22 dUTPase formed thin stacked rectangular crystals. Amino-terminal histidine-tagged dUTPases did not crystallize but formed aggregates. Glycyl-seryl-tagged dUTPases yielded cubic *P*2_1_3 IL-3A crystals with unit-cell parameter *a* = 105.68 Å and hexagonal *P*6_3_ Mu-22 crystals with unit-cell parameters *a* = 132.07, *c* = 53.45 Å, γ = 120°. Owing to the Thr84Arg mutation, Mu-22 dUTPase had different monomer-to-monomer interactions to those of IL-3A dUTPase.

## Abstract 2

Algal *Chlorella* virus IL-3A deoxyuridine triphosphatase and its Glu81Ser plus Thr84Arg-mutated derivative, Mu-22, were crystallized using the hanging-drop vapor-diffusion method. Glycyl-seryl-tagged dUTPases yielded cubic and hexagonal crystals for IL-3A and Mu-22, respectively.

## 1. Introduction

The optimal temperature for enzyme activity is determined by a trade-off between structural stability and chemical reactivity and is often similar to the physiological conditions of the source organism (Vieille & Zeikus, 2001 ▶). Previously, we have used the monomeric protein ribonuclease A (RNase; Kadonosono *et al.*, 2003 ▶; Chatani *et al.*, 2002 ▶) and the homodimeric protein 3-isopropylmalate dehydro­genase (IMD; Imada *et al.*, 1991 ▶; Moriyama *et al.*, 1995 ▶; Hori *et al.*, 2000 ▶) to study the relationship between temperature and enzyme activity. These studies showed that the size of the active-site cavity influences enzyme stability. In RNase, the internal size of the active-site cavity is inversely correlated with stability (Chatani *et al.*, 2002 ▶). In IMD, a single mutation, Gly240Ala, within the active-site cavity resulted in loss of enzyme thermostability; the optimal temperature decreased owing to the loss of hydrophobic interactions of the sub­units with the enlarged internal cavity (Moriyama *et al.*, 1995 ▶). More recently, we have studied the relationship between temperature and enzyme activity using the homotrimeric enzyme deoxyuridine pyro­phosphatase (dUTPase; Zhang *et al.*, 2005 ▶; Bajaj & Moriyama, 2007 ▶).

dUTPase (EC 3.6.1.23) is a ubiquitous enzyme (Cedergren-Zeppezauer *et al.*, 1992 ▶) that catalyzes the hydrolysis of dUTP to diphosphate and dUMP, the substrate for thymidylate synthetase in dTTP biosynthesis (Phan *et al.*, 2001 ▶). The first dUTPase crystal structure to be resolved was that of *Escherichia coli* (Cedergren-Zeppezauer *et al.*, 1992 ▶). Since then, the structure of human dUTPase (Mol *et al.*, 1996 ▶) has been identified, as have those of human pathogens, including tuberculosis (Varga *et al.*, 2008 ▶; Vertessy & Toth, 2009 ▶). dUTPase controls the pyrimidine-nucleotide pool balance and hence is involved in cell death (Mashiyama *et al.*, 2008 ▶). Recently, dUTPase has received further clinical attention as compromising dUTPase activity with oxaliplatin (*via* tumor protein p53) has been found to increase the efficiency of fluorouracil (5-FU) inhibition of thymidylate synthetase (Wilson *et al.*, 2009 ▶). Physicochemical studies (Takacs *et al.*, 2004 ▶) and structural insights into drug design have been reported (Samal *et al.*, 2007 ▶).

The algal *Chlorella* viruses PBCV-1 and IL-3A have dUTPases with different optimal temperatures (323 K for the former and 310 K for the latter; Zhang *et al.*, 2005 ▶). We previously generated Mu-22 dUTPase by mutating two IL-3A residues to the corresponding residues from PBCV-1 dUTPase (Zhang *et al.*, 2005 ▶). The presence of these two mutations (Glu81Ser and Thr84Arg), creating the derivative Mu-22 dUTPase, led to an increase in the optimal temperature of the enzyme to 328 K (Zhang *et al.*, 2005 ▶). Here, we conduct structural studies of dUTPases from *Chlorella* viruses in order to elucidate the molecular mechanisms behind the differences in optimal temperature.

## 2. Methods and results

**Figure 1:**
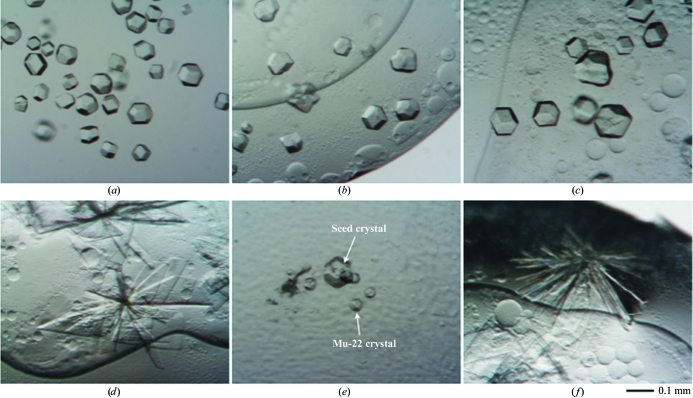
Crystallization of T7-His-tagged dUTPases. (*a*) Apo IL-3A. (*b*) Soaking of apo IL-3A in dUDP. (*c*) Cocrystallization of IL3A with EDTA. (*d*) Cocrystallization of IL3A with dUDP. (*e*) Macroseeding crystallization of Mu-22 using apo IL-3A. (*f*) Cocrystallization of Mu-22 dUTPase with dUDP.

The IL-3A, Mu-22 and PBCV-1 dUTPases had a T7-epitope tag (MASMTGGQQMGRGSEF) at the N-terminus plus a histidine tag (LEHHHHHH) at the C-terminus (Zhang *et al.*, 2005 ▶). These enzymes were placed in a solution containing 50 m*M* NaH_2_PO_4_, 50 m*M* NaCl and 100 m*M* imidazole. Initial crystallization trials were performed using previously reported conditions (Vertessy & Toth, 2009 ▶). Briefly, the hanging-drop vapor-diffusion method was per­formed using a MembFac matrix (Hampton Research, Aliso Viejo, California, USA) with a 2 µl drop and 500 µl reservoir at room temperature (approximately 298 K). An initial ragged rock-like crystal was found in a droplet containing 50 m*M* sodium citrate tribasic dihydrate pH 5.6 and 10%(*w*/*v*) 2-propanol. After refining the crystallization conditions, we obtained polyhedral single crystals by using a 8.2 mg ml^−1^ protein matrix and a reservoir containing 12%(*w*/*v*) polyethylene glycol (PEG) 4000, 10%(*w*/*v*) 2-propanol and 0.3 *M* sodium citrate pH 5.65 (Fig. 1 ▶ *a*). All crystallizations were performed for one week.

The tagged IL-3A dUTPase was subjected to diffraction studies after being soaked in 2.5 m*M* deoxyuridine diphosphate (dUDP; Fig. 1 ▶ *b*) as previously described for plant dUTPase (Bajaj & Moriyama, 2007 ▶). We used the flash-freezing method for crystal mounting with 20%(*v*/*v*) glycerol, 12%(*w*/*v*) PEG 4000, 10%(*v*/*v*) 2-­propanol and 0.23 *M* sodium citrate pH 8.0. A complete data set was collected from a single crystal using Cu *K*α radiation (wavelength 1.542 Å) from a generator operating at 40 kV and 20 mA. The diffraction images were indexed, integrated and scaled using *HKL*-2000 (HKL Research Inc.; Otwinowski & Minor, 1997 ▶) and found to diffract to a resolution of up to 3.0 Å. Crystal parameters and data-collection and structure-refinement statistics are summarized in Table 1 ▶.

Preliminary structural analysis was performed using the molecular-replacement method with chain *A* of human dUTPase as a template (PDB code 1q5h; Mol *et al.*, 1996 ▶) and assuming the presence of two monomers per asymmetric unit. The structure was determined after repetitive calculations and visual inspections and contained 127 and 125 visible residues out of 143 for monomers *A* and *B*, respectively. The N-terminal and C-terminal residues were not visible in the electron-density map. The refined structure yielded an *R* factor of 0.23 for data from 30.50 to 3.0 Å resolutiom. This structure has been deposited in the Protein Data Bank with PDB code 3ca9. In addition, to remove the divalent cations, crystallization of apo IL-­3A dUTPase was performed in ethylenediaminetetraacetic acid (EDTA) and the same crystals were obtained (Fig. 1 ▶ *c*).

Cocrystallization of IL-3A dUTPase with dUDP was performed using a droplet containing 9.0 mg ml^−1^ protein and 2.6 m*M* dUDP and a reservoir containing 11%(*w*/*v*) PEG 4000, 10%(*w*/*v*) 2-pro­panol and 0.3 *M* sodium citrate pH 5.65. This yielded only thin stacked prismatic crystals (Fig. 1 ▶ *d*). Diffraction experiments on the X6A beamline of the National Synchrotron Light source showed that the crystal belonged to the orthorhombic space group *P*222, with unit-cell parameters *a* = 81.0, *b* = 96.2, *c* = 132.8 Å, and diffracted to 3.5 Å resolution.

Although crystallization of apo Mu-22 dUTPase was not success­ful, tiny polyhedral crystals were formed after macroseeding with apo cubic IL-3A dUTPase (Fig. 1 ▶ *e*). However, these crystals were unstable and repeatedly disappeared during a time frame of hours, making them useful only for studying initial crystal growth. Cocrystallization of Mu-22 dUTPase and dUDP was performed using a droplet containing 0.57 m*M* (approximately 8.2 mg ml^−1^) protein and 1.14 m*M* dUDP and a reservoir containing 10%(*w*/*v*) PEG 4000, 10%(*w*/*v*) 2-propanol and 0.3 *M* sodium citrate pH 5.6. This yielded thin stacked rectangular crystals (Fig. 1 ▶ *f*) that gave only a few diffraction spots after 30 s of exposure on the X6A beamline.

PBCV-1 dUTPase crystallization was unsuccessful because of aggregation. N-terminally His-tagged IL-3A and Mu-22 dUTPases were constructed and subjected to crystallization. However, heavy precipitation was observed throughout the initial crystallization screens. IL-3A, Mu-22 and PBCV-1 dUTPases were then expressed as glutathione *S*-transferase (GST) fusion proteins using the pGEX-2T vector (GE Healthcare, Piscataway, New Jersey, USA). This allowed higher levels of protein expression in *E. coli*. Purification using GST-Sepharose column chromatography followed by thrombin cleavage produced glycyl-seryl-dUTPases (143 amino acids). IL-3A and Mu-22 dUTPase protein solutions were concentrated to 0.7 m*M* (approximately 10 mg ml^−1^) protein. dUDP at 2 m*M* in a buffer containing 50 m*M* sodium phosphate, 50 m*M* NaCl and 1.0%(*v*/*v*) glycerol pH 8.0 was added. The hanging-drop vapor-diffusion method was used to crystallize the dUTPases. IL-3A dUTPase formed thin rod-shaped crystals after 16 h when a reservoir solution containing 20%(*w*/*v*) PEG 1450 and 5 m*M* MgCl_2_ was used. Mu-22 dUTPase formed thick short hexagonal crystals after 14 h when a reservoir solution containing 15%(*w*/*v*) PEG 1500 was used. Thrombin cleavage of the PBCV-1 fusion protein resulted in multiple fragments and no pure protein.

**Figure 2:**
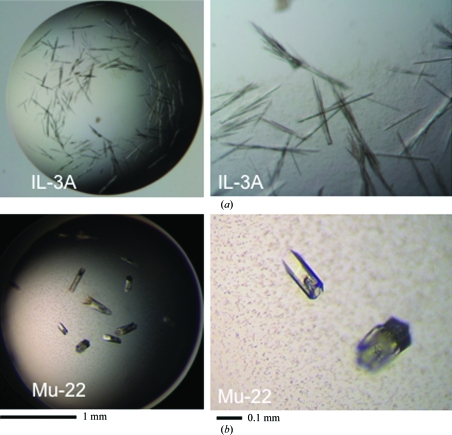
Crystals of glycyl-seryl dUTPases from IL-3A (*a*) and Mu-22 (*b*).

Preliminary X-ray studies indicated that the needle-like IL-3A dUTPase crystals consisted of two monomers and belonged to the cubic space group *P*2_1_3, with unit-cell parameter *a* = 105.68 Å (Table 1 ▶; Fig. 2 ▶ *a*; PDB code 3c2t). Coupled Mu-22 dUTPase crystals consisted of four monomers and belonged to the hexagonal space group *P*6_3_, with unit-cell parameters *a* = 132.07, *c* = 53.45 Å, γ = 120° (Table 1 ▶; Fig. 2 ▶ *b*; PDB code 3c3i).

**Figure 3:**
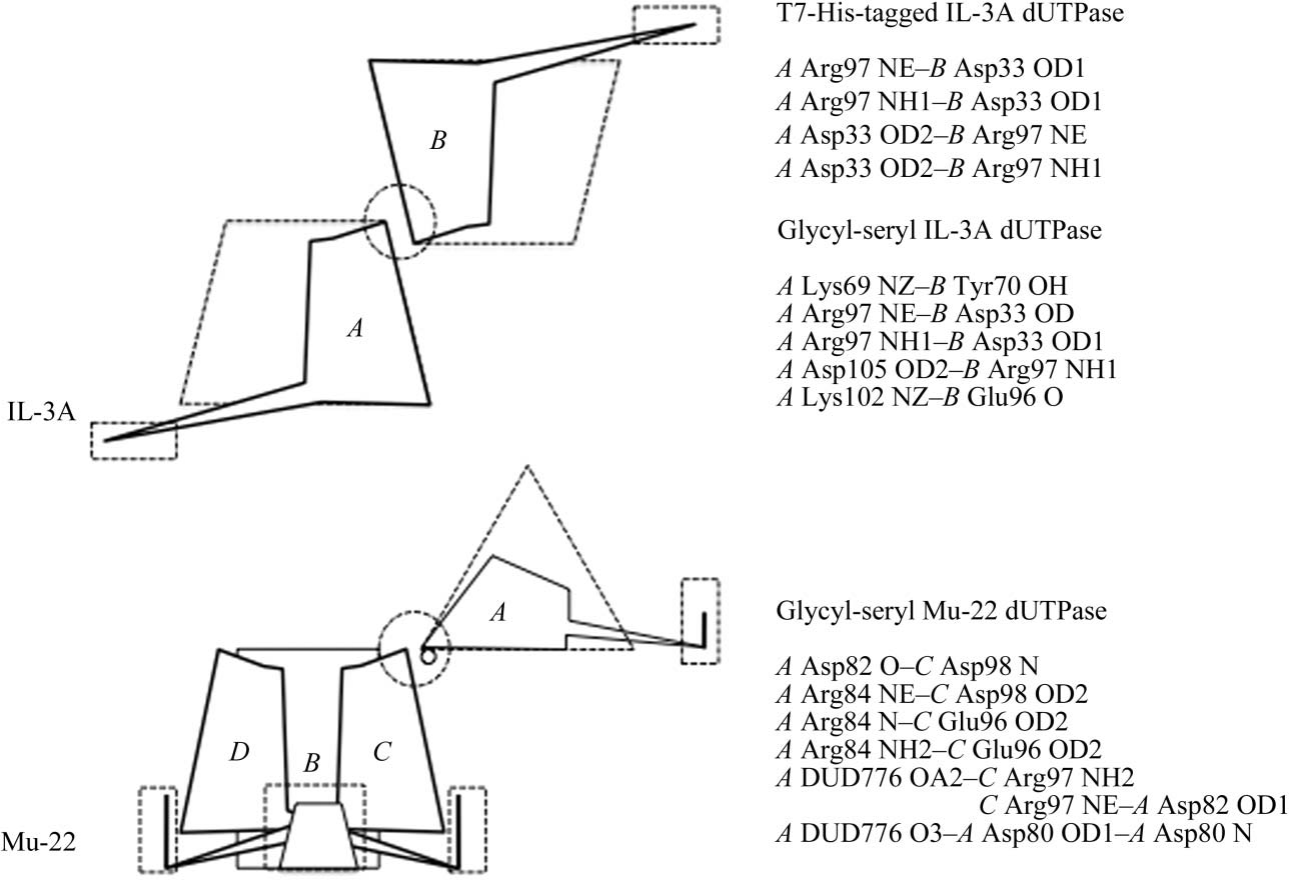
Monomer contacts in an asymmetric unit. (*a*) IL-3A dUTPase. Two monomers were seen. Dotted trapezoids represent a trimer molecule. Solid figures labeled *A* and *B* represent the two monomers (chains). Polar contacts between monomers *A* and *B* (dotted circle) are listed on the right. (*b*) Mu-22 dUTPase. One trimer and one monomer were seen. Solid figures labeled *A*, *B*, *C* and *D* represent the monomers. Monomer *A* and the trimer composed of monomers *B*, *C* and *D* are connected by dUDP (small solid circle). Polar contacts between monomer *A* and trimer *BCD* (dotted circle) are listed on the right. Dotted squares indicate the locations of the C-termini.

*Arabidopsis* dUTPase crystals consist of one asymmetric trimer (PDB code 2p9o; Bajaj & Moriyama, 2007 ▶), as do those of human dUTPase (PDB code 1q5h; Mol *et al.*, 1996 ▶); these crystals belong to the orthorhombic space groups *P*2_1_2_1_2_1_ and *P*2_1_2_1_2, respectively. Two neighboring trimers in a unit cell make a head-to-tail contact along the *xy* plane in the *Arabidopsis* and human dUTPase crystals. In human dUTPase, one of the three His-tag tails contributes to this inter-trimer interaction, whereas the other two His tags were not visible. Both the T7-His-tagged IL-3A dUTPase and the glycyl-seryl-tagged protein have the same crystal packing, but this packing differs from those of *Arabidopsis* and human dUTPases. This consists of two trimers aligned head-to-head in a 2_1_ manner held to two monomers by polar contacts (Fig. 3 ▶ *a*). The two trimer arrays are connected *via* a short antiparallel β-sheet between residues 120 and 125.

Although we used very similar crystallization conditions, Mu-22 dUTPase had different monomer-to-monomer interactions from those of IL-3A dUTPase owing to the mutation Thr84Arg (Fig. 3 ▶). As a result, glycyl-seryl-tagged Mu-22 dUTPase consists of a dUDP molecule located between a monomer and trimer. The dUDP provides intermonomer interactions *via* polar contacts (Fig. 3 ▶ *b*). Instead of the head-to-head interaction found in IL-3A, side interactions were formed in Mu-22 (Fig. 3 ▶ *b*). The dUTPase molecules form a blunted pyramid shape and thus the molecules do not fill the space perfectly, creating a central hole in the crystal.

## Figures and Tables

**Table 1:** Summary of crystallographic data

dUTPase	T7-His-tagged IL-3A	Gly-Ser IL-3A	Gly-Ser Mu-22
PDB code	3ca9	3c2t	3c3i
Crystallization	Soaking with dUDP	Cocrystallization with dUDP	Cocrystallization with dUDP
Space group	*P*2_1_3	*P*2_1_3	*P*6_3_
Unit-cell parameters (Å, °)	*a* = 105.65	*a* = 105.68	*a* = 132.07, *c* = 53.45, γ = 120.0
Interactions†	Head-to-head	Head-to-head	Side
Resolution	50.0–3.0	50–3.0	50–2.4
Total reflections	84696	80123	88640
Unique reflections	8218	8112	20383
Completeness (%)	99.9	99.4	96.6
Redundancy	10.3	9.9	4.4
*R*_merge_	0.10	0.10	0.06
*I*/σ(*I*)	13.4	13.2	21.4
Refinement (Å)	30.50–3.0	33.42–3.0	33.61–3.0
Completeness (%)	100.0	97.8	99.0
*R*_work_	0.232	0.245	0.193
*R*_free_	0.307	0.285	0.263
*B*_average_ (Å^2^)	30.0	30.3	28.0
No. of subunits in ASU	1 + 1	1 + 1	1 + 3
Residues determined			
Chain *A*	−2–125	1–125	2–128
Chain *B*	0–125	1–124	2–137
Chain *C*			2–137
Chain *D*			2–131
Protein atoms	1909	1864	3939

† Observed intermonomer interactions in crystals. Details are given in Fig. 3 ▶.
